# Risk of imbalanced glycemic pattern: diagnostic content validity

**DOI:** 10.1590/1980-220X-REEUSP-2024-0298en

**Published:** 2025-03-31

**Authors:** Letícia Mattos Gonçalves, Rafael Oliveira Pitta Lopes, Eduardo da Silva Gomes, Thamiris da Silva e Silva, Rosane Barreto Cardoso, Marcos Antônio Gomes Brandão

**Affiliations:** 1 Universidade Federal do Rio de Janeiro, Escola de Enfermagem Anna Nery, Rio de Janeiro, RJ, Brazil.

**Keywords:** Validation Study, Diabetes Mellitus, Glycemic Control, Nursing Diagnosis, Standardized Nursing Terminology, Estudo de Validação, Diabetes Mellitus, Controle Glicêmico, Diagnóstico de Enfermagem, Terminologia Padronizada em Enfermagem, Estudio de Validación, Diabetes Mellitus, Control Glucémico, Diagnóstico de Enfermería, Terminología Normalizada de Enfermería

## Abstract

**Objective:**

To assess evidence of content validity of the diagnostic proposition “Risk of imbalanced glycemic pattern”.

**Method:**

Content validity study based on collective wisdom, with a quantitative approach. Sample composed of 51 participants who assessed the relevance and accuracy of 13 risk factors, 10 at-risk populations, and 16 associated conditions. For analysis, the content validity index was used, excluding diagnostic components that reached a value equal to or less than 0.5.

**Results:**

The diagnostic label and its definition achieved a content validity index of 0.98 and 0.94, respectively. The risk factors with the highest rates were inadequate blood glucose monitoring, inadequate knowledge of disease management, inadequate regularity of meal consumption, obesity, and overweight. No diagnostic component was excluded, as all of them presented evidence of content validity.

**Conclusion:**

The diagnostic proposition Risk of imbalanced glycemic pattern reached adequate values for consideration of evidence of content validity.

## RESUMO

**Objetivo:**

Avaliar a evidência de validade de conteúdo da proposição diagnóstica “Risco de padrão glicêmico desequilibrado”.

**Método:**

Estudo de validade de conteúdo baseado na sabedoria coletiva, de abordagem quantitativa. Amostra composta por 51 participantes que avaliaram a relevância e precisão dos 13 fatores de risco, 10 populações em risco e 16 condições associadas. Para análise, foi utilizado o índice de validade de conteúdo, sendo excluídos os componentes diagnósticos que tenham atingido valor igual ou inferior a 0,5.

**Resultados:**

A etiqueta diagnóstica e sua definição alcançaram um índice de validade de conteúdo de 0,98 e 0,94, respectivamente. Os fatores de risco com maiores índices foram monitoramento inadequado da glicemia, conhecimento inadequado do manejo da doença, regularidade inadequada do consumo de refeições, obesidade e sobrepeso. Nenhum componente diagnóstico foi excluído, pois todos apresentaram evidência de validade de conteúdo.

**Conclusão:**

A proposição diagnóstica Risco de padrão glicêmico desequilibrado atingiu adequados valores para a consideração de evidência de validade de conteúdo.

## RESUMEN

**Objetivo:**

Evaluar la evidencia de validez de contenido de la propuesta diagnóstica “Riesgo de patrón glucémico desequilibrado”.

**Método:**

Estudio de validez de contenido basado en la sabiduría colectiva, con un enfoque cuantitativo. Muestra compuesta por 51 participantes que evaluaron la relevancia y precisión de 13 factores de riesgo, 10 poblaciones en riesgo y 16 afecciones asociadas. Para el análisis se utilizó el índice de validez de contenido, excluyendo los componentes diagnósticos que alcanzaron un valor igual o menor a 0,5.

**Resultados:**

La etiqueta diagnóstica y su definición alcanzaron un índice de validez de contenido de 0,98 y 0,94, respectivamente. Los factores de riesgo con tasas más altas fueron el control inadecuado de la glucemia, conocimientos inadecuados sobre el manejo de la enfermedad, regularidad inadecuada en el consumo de comidas, obesidad y sobrepeso. No se excluyó ningún componente diagnóstico, ya que todos presentaron evidencia de validez de contenido.

**Conclusión:**

La propuesta diagnóstica Riesgo de patrón glucémico desequilibrado alcanzó valores adecuados para ser considerada evidencia de validez de contenido.

## INTRODUCTION

The glycemic pattern is a construct used in health to characterize and understand glycemic homeostasis in humans, being defined as a variable response that informs the nature of the regularity of serum glucose oscillations that occur on the same day (intraday) and on subsequent days (interday)^(1)^. The purpose of determining glycemic targets for people with Diabetes Mellitus (DM) is to achieve balanced glycemic patterns and, to this end, continuous adjustments and adaptations during the life and coexistence with the disease are necessary, with monitoring by the health team being essential, especially focused on habits, styles and life processes. The socio-environmental context must be considered to ensure individualization of treatment, meeting each individual’s needs, preferences, and living conditions^(2)^.

The potential imbalance of the glycemic pattern was theorized in the medium range, with the presentation of relations with factors that can be modified independently by nursing interventions and their relations with factors that cannot be modified by independent nursing actions. Among the factors subject to independent nursing actions, we highlight those related to aspects of body weight, food/nutrition, knowledge, harmful habits, cognition, physical activity, adherence behavior, and access to health care^(1)^.

Vulnerability to an imbalance in the glycemic pattern can be considered a human response, characterized as an at-risk Nursing Diagnosis (ND). At-risk NDs are defined as “susceptibility to developing, in the future, an undesirable human response to health conditions/life processes”^(3)^. The Risk for an Imbalanced Glycemic Pattern (*RIGP*) originated from the inquiry about the adequacy of the “unstable” judgment axis present in the ND “Risk for unstable glycemia” and was defined as “susceptibility to recurrent amplitudes of fluctuations in the blood glucose level out of the desirable range, which occur throughout the day and/or on different days, with standard deviation of glucose measurements below 50 mg/dL and/or coefficient of variation below 36%”^(4)^. Its development was based on a construct analysis^(4)^ and was subjected to the *Diagnosis Development Committee (DDC)* from the NANDA-International^®^ (NANDA-I^®^), so far not being included in the diagnostic taxonomy. Unlike the recently included ND in the taxonomy, Risk for ineffective self-management of glycemic pattern (00489), which is oriented towards self-management, the ND RIGP is oriented towards the imbalance of the focus, namely, the glycemic pattern.

According to the criteria established by NANDA-I^®^, it is understood that the ND RIGP presents evidence of first-level validity, such as validity in the theoretical-causal scope. Furthermore, despite the theoretical and conceptual advances obtained through mid-range theorization, the advance in the levels of evidence for the RIGP diagnosis requires second-level validity evidence, such as content validity, which is essential for improving a diagnostic proposal^(3)^. Conducting this study on the content validity of the RIGP allows us to verify how representative the conceptual components of the diagnosis are for their clinical content domain^(3)^ and allows their improvement. Therefore, the advancement in the level of evidence of the RIGP strengthens the diagnostic proposal for inclusion in the standardized language system. Its inclusion will favor the identification of RIGP in clinical practice, which will potentially contribute to the prevention of health complications and target organ damage in people with DM, since these clinical outcomes are caused, among other factors, by the maintenance of imbalanced glycemic patterns. Thus, this research explores an uninvestigated dimension of the RIGP, however, which is essential both for the diagnostic utility of the standardized language system and for its use in clinical practice. Therefore, the objetive of the study is to assess evidence of content validity of the diagnostic proposition “Risk of imbalanced glycemic pattern”.

## METHOD

### Design of Study

Diagnostic content validity study was conducted according to modality 2.2.2 of potential diagnostic content validity of the NANDA-I taxonomy^®(3)^, to advance the levels of evidence of the ND RIGP. The theoretical reference for defining content validity was collective wisdom^(5)^. The study followed the recommendations proposed by *Guidelines for Reporting Reliability and Agreement Studies* (GRASS)^(6)^.

### Local

All stages of this study were carried out virtually.

### Population and Selection Criteria

The study population consisted of nurses. The process of recruiting potential participants took place in the directory of research groups in Brazil Lattes on the portal of the National Council for Scientific and Technological Development (CNPq). Brazilian research groups that had at least one of the following terms in their title and/or line of research and/or keyword were investigated: “Systematization of Nursing Care” (SNC); “Nursing Process” (NP); ND, and DM. Furthermore, to increase the capture of potential participants, the snowball sampling technique was used. Then, referral chains were used, in which the initial study participants indicated other participants who fit the profile of interest.

The criteria for selecting participants were: presenting studies on at least one of the following themes: SNC, ND, DM and NP; participating or having participated in research groups that present in their title and/or line of research and/or keyword at least one of the following terms: SNC, ND, DM and NP. Those who met the first criterion and at least one of the other criteria were included in the study. Those who completed the instrument partially or incorrectly were considered lost for the research.

Clinical and academic experience was assessed to characterize the nurses’ level of expertise^(7)^. This characterization was carried out with the purpose of meeting the criteria of the collective wisdom approach and predictive diversity of the study.

### Sample Definition

The formula used to calculate the sample was n_0_ = (Z_1-α/2_.s/ϭ)2, where: Z1-α/2 refers to the confidence level to be adopted; s represents the standard deviation; ϭ corresponds to the sampling error^(5)^. In this study, to determine the minimum number of experts, the following parameters were designated: 95% confidence level (Z_1-α/2_ is equal to 1.96); standard deviation of 0.17 and, sampling error of 0.05. Thus, we have: n_0_ = 45 participants. Considering that the Content Validity Index (CVI) distributions may be asymmetric, a likely loss in the non-parametric test^(5)^, then the sample size was adjusted by an increase of 5% (48 participants). A total of 568 invitations were sent to potential participants. However, 51 responses were accepted and none of the participants were discontinued.

### Data Collection

Potential participants received an invitation to participate via email, containing a link to access the electronic form with the data collection instrument, which was divided into six sections, namely: 1) Free and Informed Consent Form (FICF); 2) categorization of experts; 3) filling guidelines, analysis of the diagnostic label, and risk factors; 4) analysis of the population at risk; 5) analysis of associated conditions and 6) acknowledgments. The deadline for return was a maximum of 20 days after receipt of the instrument. Data were collected from June 2023 to August 2023.

The variables characterizing the participants were: clinical experience (involving the time of work with the theme of ND and/or DM) and academic experience (related to the time of participation in a research group on nursing terminology and/or DM and scientific knowledge). Using the characterization data, participants were classified into five levels of expertise^(7)^: (1) Novice, (2) Advanced Beginner, (3) Competent, (4) Proficient, and (5) Expert.

The variables of interest in the study were the criteria of relevance, precision, and clarity. The diagnostic label, its definition, and each risk factor, associated condition, and population at risk of the ND RIGP were assessed for relevance. Accuracy was assessed in the constitutive and operational definitions of each risk factor, associated condition, and population at risk of the ND RIGP. Both relevance and accuracy were assessed using a five-point Likert scale (completely disagree, partially disagree, indifferent, partially agree and completely agree). Clarity was assessed through the areas for comments and suggestions. Judges assessed the clarity of the diagnostic label, definition of the diagnosis, as well as constitutive and operational definitions of each risk factor, associated condition, and population at risk. When the judges considered that the definitions already met the criterion of clarity, they left the space blank.

The constitutive definitions were constructed from Portuguese language dictionaries, DeCS, MeSH, and the glossary of the Brazilian Ministry of Health. Regarding the elaboration of operational definitions, primary studies of construct analysis that gave rise to the diagnosis and guidelines of different professional societies such as the Brazilian Diabetes Society (SBD), *American Diabetes Association* (ADA), *American Heart Association* (AHA), and *Kidney Disease Improving Global Outcomes* (KDIGO) were used.

### Data Analysis and Treatment

Data were organized in a spreadsheet in *software Microsoft Office Excel 365.* For the treatment of descriptive data, the following was used: *software Statistical Package for the Social Sciences* (IBM-SPSS) version 24. And for the inferential analytical treatment, the statistical software R version 4.1.1 was used. Descriptive statistical analysis was performed in table form, where the frequencies and percentages of participants’ responses and variables in the study were recorded. To analyze the content validity of the ND RIGP, the CVI was calculated. The weighted estimates of the mean and median of the evaluations were calculated and from them it was decided that the weighted median CVI estimate would be used, according to the normality or not of the distribution of these estimates.

After initial data assessments, the I-CVI indicators (indicators for each item) and S-CVI/Ave (average of the content validity indexes for all indexes of the scale/*Average extracted variance*) were calculated. To calculate the individual I-CVI, the experts’ responses “partially agree” (4) and “totally agree” (5) were added and the sum divided by the total number of responses obtained in each item^(8)^. Therefore, the formula used was I-CVI = number of responses “partially agree” or “totally agree”/total number of responses. The S-CVI was calculated using the average CVI among all items analyzed^(8)^. The values obtained in the I-CVI guided decisions on revisions or rejections of diagnostic components^(8,9)^. Regarding the measurement of the general CVI of the ND, all I-CVIs were added and divided by the total number of items. Evidence of satisfactory content validity was considered to be an I-CVI equal to or greater than 0.9; evidence of minimum acceptable content was an I-CVI value between 0.78 and 0.89, and candidate for review of the diagnostic component in cases where the I-CVI value was less than 0.78. Items with I-CVI equal to or less than 0.5 were excluded as they were unacceptable^(9)^. The criterion for content validity from the S-CVI was a value greater than or equal to 0.90^(9)^. Finally, the experts’ agreement was assessed using the Gwet Agreement Test^(10)^. In this work, a significance level of 5% and 95% confidence were considered.

The criteria for clarity of conceptual and operational definitions were qualitatively assessed by the judges, who were able to make observations and suggest changes when they deemed necessary. The suggestions were checked by the authors and relevant changes were made to the definitions.

### Ethical Aspects

The research was carried out in accordance with resolution no. 466/2012 and circular letter no. 2/2021 – CONEP/SECNS/MS, dated February 24, 2021. This study was approved by the Research Ethics Committee, according to opinion no. 5.812.414, CAAE 65270922.5.0000.5238. All participants in this study signed the informed consent form.

## RESULTS

Among the 51 participants, there was a predominance of women (80%), with an average age of 35.47 years, masters (49%) and PhDs (35%), from the Southeast (55%) and Northeast (29%) regions of Brazil, having worked in the last 12 months in educational institutions (37%). Professionals who taught subjects on the topic of nursing terminology represented 78% and, on DM, 55% of the participants. Regarding the level of expertise, advanced beginners (45%) and competent (25%) predominated. The average time spent working in research groups on terminologies and/or DM was 2 years; the training time was 11.63 years and the median was 12 years. The time of practice in the area of terminology and/or DM had an average of 7.63 years with a median of 6.5 years.

The overall CVI of the diagnosis was 0.94. No diagnostic component was excluded, as all of them presented evidence of content validity. Regarding the validity of the diagnostic label and its definition, the I-CVI values were 0.98 and 0.94 respectively, producing the S-CVI of 0.96 with a Gwet value of 0.658 (0.505; 0.811) with p-value < 0.0001. Suggestions for clarity of diagnostic labeling were not met because they disregarded the conceptual definitions of the judgment axis of the NANDA-I taxonomy^®^. Suggestions for changes to the definition were accepted for simplification purposes, resulting in the final text for the definition: “susceptibility to recurring fluctuations in glucose levels out of the desired target throughout the day and/or on subsequent days”. All risk factors were considered valid regarding the relevance and accuracy of the constitutive and operational definitions in I-CVI values that ranged from 0.88 to 1.00, as shown in Table 1.

**Table 1:** Distribution of experts’ responses regarding the risk factors of the nursing diagnosis “Risk for imbalanced glycemic pattern” regarding the relevance and precision of the constitutive and operational definitions – Rio de Janeiro, RJ, Brazil, 2023.

Risk factors	I-CVI relevance	I-CVI precision constitutive definition	I-CVI precision operational definition
1. Excessive stress	0.96	0.92	0.98
2. Cognitive Dysfunction	0.92	0.94	0.88
3. Excessive alcohol consumption	1.00	0.98	0.88
4. Excessive daily physical exercise	0.90	0.88	0.94
5. Inadequate follow-up of the treatment regimen	0.96	0.92	0.96
6. Inadequate blood glucose monitoring	1.00	0.98	0.96
7. Inadequate knowledge of disease management	1.00	0.96	0.98
8. Inadequate management of food quantity	0.98	1.00	0.96
9. Inadequate regularity of meal consumption	1.00	1.00	0.98
10. Obesity	1.00	1.00	1.00
11. Overweight	1.00	1.00	1.00
12. Smoking	0.92	0.92	0.90
13. Body weight below the ideal weight range for age and sex	0.96	0.98	0.98
**Risk factors**	**S-CVI/AVE**	**Gwet value**	**p-value**
Relevance	0.97	0.807 (0.748; 0.867)	<0.001
Accuracy Constitutive Definition	0.96	0.751 (0.673; 0.830)	<0.001
Operational Definition Accuracy	0.95	0.746 (0.670; 0.823)	<0.001

AVE: *Average extracted variance*; I-CVI: Item Content Validity Index; S-CVI: Average of Content Validity Indexes.

In the diagnostic element of populations at risk, participants considered populations of individuals of African descent, indigenous people, the older people, and blood glucose values below the normal limit in the preoperative period to be minimally acceptable. The only constitutive and operational definitions with minimally acceptable precision were populations of individuals of African descent, indigenous people, and preoperative lower limit of normal blood glucose values. The other populations were considered to have satisfactory content validity criteria and are shown in Table 2 with the I-CVI values.

**Table 2:** Distribution of expert responses regarding populations at risk for the nursing diagnosis “Risk for imbalanced glycemic pattern” regarding the relevance and accuracy of the constitutive and operational definitions – Rio de Janeiro, RJ, Brazil, 2023.

Populations at risk	I-CVI relevance	I-CVI precision constitutive definition	I-CVI precision operational definition
1. Lower limit of normal blood glucose values preoperatively	0.86	0.86	0.88
2. Older people	0.86	0.92	0.92
3. Individuals on social vulnerability	1.00	0.98	0.98
4. Individuals with low educational level	0.94	0.90	0.90
5. Individuals of African descent	0.78	0.84	0.84
6. Indigenous individuals	0.78	0.84	0.88
7. Individuals with prolonged duration of diabetes	0.92	0.90	0.92
8. Individuals with asymptomatic hypoglycemia	0.98	1.00	0.98
9. Individuals with a history of hypoglycemia	0.98	1.00	0.98
10. Individuals voluntarily experience a prolonged fasting period	0.96	0.96	0.96
**Populations at risk**	**S-CVI/AVE**	**Gwet value**	**p-value**
Relevance	0.91	0.689 (0.605; 0.772)	<0.001
Accuracy Constitutive Definition	0.93	0.714 (0.678; 0.801)	<0.001
Operational Definition Accuracy	0.93	0.721 (0.634; 0.805)	<0.001

AVE: *Average extracted variance*; I-CVI: Item Content Validity Index; S-CVI: Average of Content Validity Indexes.

When analyzing the associated conditions, all diagnostic components were validated in relevance and accuracy in values ranging from 0.86 to 1.00 (see Table 3).

**Table 3:** Distribution of experts’ responses in relation to the associated conditions of the nursing diagnosis “Risk for imbalanced glycemic pattern” regarding the relevance and precision of the constitutive and operational definitions – Rio de Janeiro, RJ, Brazil, 2023.

Associated conditions	I-CVI relevance	I-CVI precision constitutive definition	I-CVI precision operational definition
1. Altered glycated hemoglobin	0.96	0.96	0.96
2. Assessment of altered homeostatic model for insulin resistance score	0.94	0.94	0.94
3. Cardiovascular Disease	0.94	0.96	0.92
4. Cerebrovascular disorders	0.90	0.96	0.96
5. Decreased serum albumin level	0.90	0.92	0.92
6. Diabetic retinopathy	0.98	0.98	0.98
7. Increased morbidity	0.90	0.92	0.94
8. Infections	0.94	0.96	0.96
9. Kidney diseases	1.00	1.00	1.00
10. Liver diseases	0.96	0.98	0.98
11. Mental disorder	0.90	0.92	0.92
12. Neoplasms	0.94	0.96	0.94
13. Peripheral neuropathy	0.94	0.96	0.94
14. Pharmaceutical preparations	0,86	0.92	0.88
15. Polypharmacy	0.92	0.94	0.92
16. Individuals with ulcers in the lower limbs	0.86	0.90	0.90
**Associated conditions**	**S-CVI/AVE**	**Gwet value**	**p-value**
Relevance	0.93	0.803 (0.731; 0.876)	<0.001
Constitutive Definition	0.95	0.825 (0.756; 0.894)	<0.001
Operational Definition	0.94	0.821 (0.746; 0.896)	<0.001

AVE: *Average extracted variance*; I-CVI: Item Content Validity Index; S-CVI: Average of Content Validity Indexes.

Based on participants’ suggestions, writing adjustments were made to improve understanding of the diagnostic components, such as changing the name of the risk factor “Inadequate management of food quantity” to “Inadequate management of the quantity of food consumed”. In addition, adjustments were made to the constitutive definitions of the risk factors: excessive stress; excessive daily physical exercise; inadequate monitoring of the treatment regimen; inadequate monitoring of blood glucose levels; inadequate knowledge of disease management; inadequate management of the amount of food consumed and smoking; of the populations at risk: older people and individuals with prolonged duration of diabetes. Regarding the operational definitions, modifications were made to the diagnostic components: cognitive dysfunction; excessive alcohol consumption; older people; individuals with social vulnerability, and indigenous individuals.

## DISCUSSION

High values of the general content validity index, as well as for the diagnostic label and its definition, are consistent with an inference of adequacy of representativeness between the construct of risk of imbalanced glycemic pattern (generated at a conceptual level) and the human response already observed by the participants (at a clinical practice level). When proposing a new ND, the label and definition have to represent the human response for which the nurse can implement autonomous nursing interventions^(3)^. And more than that, the correspondence between the diagnostic concept and the clinical elements of the diagnosis must be sufficiently robust so that the ND is not just a conceptual elaboration without correspondence in the empirical expressions of the human response. Thus, the results obtained are evidence of the existence of the RIGP beyond the theoretical-conceptual domain.

Suggestions for changes to the diagnostic label, exclusively on the judgment axis, were not considered because they contradict the conceptualization of the middle-range theory on the term “imbalanced”. This term was selected because it is the judgment about the undesirable changes occurring in the glycemic pattern. An “imbalanced” judgment refers to the lack of proportion in corresponding values^(3)^. For a glycemic pattern, the imbalance is characterized by the disproportion between the set of observed glycemic measurements that go beyond the ranges and parameters of glycemic values established as goals for the individual living with DM.

The new NANDA-I^®^ diagnosis (00489) *Risk for ineffective glucose pattern self-management* is notably a ND oriented towards dealing with symptoms, treatment regimen, and lifestyle changes associated with imbalance in the glycemic pattern^(3)^. Its positioning in the health promotion domain and in the health management class makes explicit the nature of the diagnosis of directing its focus to the actions of the person who experiences the glycemic pattern and estimates a vulnerability to failure in the use of self-management strategies for the condition. In contrast, RIGP is a diagnosis primarily aimed at a person’s vulnerability to experiencing an imbalance in the glycemic ­pattern, which expands the scope of clinical judgment beyond the self-management properties of the person with DM.

National and international guidelines highlight that there are different measures to be evaluated and compared to desirable parameters and that are useful for diagnosing patterns, such as value ranges, coefficients and degrees of dispersion of a data set, to avoid health complications for people with DM^(2,11,12)^. Isolated blood glucose measurements are compared to the value ranges established as desirable according to the time of meal, such as fasting, pre- and post-prandial. Thus, the core of the glycemic standard is the evaluation of a set of measurements to be compared to coefficient values and degree of dispersion, called coefficients of variation and standard deviation of glycemia. Another measure evaluated is the verification of the time the individual remained on the target, known as *time in range*. Thus, the new proposed definition based on the experts’ judgment simplifies the elements pertinent to the glycemic pattern and the lack of equality between the values of variation and dispersion of glycemia and the corresponding recommended values (targets) on an individual basis^(1)^.

Overall, the risk factors for the RIGP showed satisfactory evidence of content validity. Some did not obtain the maximum index and the difficulties of interpretation that recursive causality factors can produce can be considered as justification. Cognitive dysfunction, for example, can cause inadequate blood glucose management^(13)^, but it may also be an effect of the course of the DM disease in the presence of other comorbidities^(13,14)^. Maintaining a balanced glycemic pattern is extremely important for preserving and improving cognitive function, with emphasis on regulating blood glucose levels associated with the cognitive health of individuals with DM^(15)^.

The risk factor of excessive daily physical exercise can be selected to understand the complexity of causal relations, and possibly, the failure to reach maximum I-CVI values. Although the benefits of physical exercise are widely recognized for several health outcomes in people with DM^(16)^, including for glycemic management^(17)^, excessive physical exercise can lead to negative outcomes such as hypoglycemia, impacting the imbalance of the glycemic pattern^(2,18)^. Therefore, physical exercise should be guided by the specific needs of each individual according to the type of diabetes, age, the activity to be performed and the presence of related health complications^(16,17,18)^.

When qualitatively analyzing the participants’ suggestions for the at-risk populations of individuals with African ancestry and indigenous individuals, we found that there was little knowledge about the scientific evidence that presents the correlation between the imbalanced glycemic pattern and individuals with African ancestry, resulting from the low rates of disease management when compared to the white population^(19)^. This consideration indirectly instills factors on social determination of health on a global scale and which should not be disregarded in clinical nursing judgment. The literature suggests that this population may no longer be vulnerable if there are culturally adapted lifestyle interventions on glycated hemoglobin (HbA1c) and fasting blood glucose in people with type 2 Diabetes Mellitus (DM2) or pre-diabetes of black African descent^(19)^. Another point is the underlying propensity for the higher prevalence of DM2 in this ethnic group, highlighting the desensitization of the insulin receptor, the favoring of insulin resistance in target tissues and the subsequent glucose intolerance^(20)^.

For the indigenous population, studies highlight sociodemographic and genetic aspects and the difficulty of accessing health care as responsible for inadequate glucose variations^(21,22,23)^. The difficulty in implementing self-management practices of blood glucose is understood, mainly due to the absence of the occurrence of DM before the colonization of these groups^(21)^. Furthermore, the change in eating habits and physical activity in the daily lives of villages stands out, leading to the consumption of processed foods due to difficulties in agriculture and food shortages^(22)^. In addition to the extrinsic factors presented here, a genetic study carried out in the indigenous Amazonian population found the presence of four genes associated with DM2 in different ethnic populations, two other genes related to complications associated with DM and the identification of a high-impact variant^(23)^.

The associated condition “individuals with ulcers in the lower limbs” reached a minimum acceptable value in the content evidence, possibly explained by recursive causality. Hyperglycemia is related to the emergence of lower limb diseases, such as peripheral arterial disease and peripheral neuropathy^(24)^. Similar to HbA1c values, major amputations and peripheral diseases in adults with DM are biomarkers of diabetic foot ulcers^(24,25)^. Consequently, inadequate blood glucose management is one of the main factors related to the incidence of foot ulcers, risk of amputation, and/or reamputation of lower limbs in individuals with DM^(26)^. However, it is also recognized that the appearance of ulcers in the lower limbs prevents the implementation of lifestyle changes necessary to maintain target blood glucose levels, such as walking, compromising the performance of physical activities^(27)^.

Another condition associated with minimum acceptable content evidence was that of pharmaceutical preparations. Despite this finding, the literature highlights the contributions of drugs widely used in DM for changes in the glycemic profile and comorbidities, such as metformin, hydrochlorothiazide, and rosuvastatin^(28)^. In addition to the pharmacological effects themselves, it is known that polypharmacy also contributes to the difficulty in maintaining target blood glucose levels, especially compromising glycemic control in the elderly and favoring the emergence of hypoglycemic events in this group^(29,30)^. It is also worth noting that interactions between antidiabetic drugs and other drugs affect the pharmacokinetics of antidiabetics, preventing the maintenance of blood glucose levels at the target level^(29,30)^ and leading to the “prescription cascade” process^(29)^.

The limitation of the study, which affects external validity, is related to the nature of the biases related to the participant’s memory and their real familiarity with the human response consistent with the RIGP. However, this limitation was minimized by the adequate sample size, by the selection of a diversity of levels of expertise in the application of the principle of collective wisdom, and by the observation of high values for the I-CVI to consider a valid element. Moreover, the limits of the proposed diagnosis for the context of people with DM are highlighted, without primarily considering the specificities of other conditions such as critical health statuses.

The study brings advances to nursing and health by producing useful evidence for estimating vulnerability to an imbalanced glycemic pattern, guiding clinical judgment, particularly that of nurses. It also encourages new research into the conceptual and clinical differentiation of the ND currently classified by NANDA-I.^®^, as well as supporting future research on different levels of evidence for the RIGP.

## CONCLUSION

The research findings present evidence of content validity of the elements of the RIGP diagnostic proposition. The use of the collective wisdom principle approach proved to be suitable for composing a sample of participants with diverse levels of qualification, training, and other variables of interest that promoted a reliable judgment of the content.

The results of this investigation do not refute the existence of the RIGP, and show that the diagnostic construct has ­evidence of validity for clinical use as an indicator of the human response that warns of susceptibility to recurring fluctuations in glucose levels outside the desirable target throughout the day and/or on subsequent days.
